# Dispersive Modeling of Normal and Cancerous Cervical Cell Responses to Nanosecond Electric Fields in Reversible Electroporation Using a Drift-Step Rectifier Diode Generator

**DOI:** 10.3390/mi14122136

**Published:** 2023-11-22

**Authors:** Mayank Kumar, Sachin Kumar, Shubhro Chakrabartty, Alwin Poulose, Hala Mostafa, Bhawna Goyal

**Affiliations:** 1 Technical Research Analyst (TRA), Electronics/Biomedical Engineering, Aranca, Mumbai 400076, Maharastra, India; mayank.kumar@aranca.com; 2 Department of Electronics and Communication Engineering, Galgotias College of Engineering and Technology, Greater Noida 201310, Uttar Pradesh, India; gupta.sachin0708@gmail.com; 3 School of Computer Science Engineering and Applications, D Y Patil International University, Pune 411044, Maharastra, India; 4 School of Data Science, Indian Institute of Science Education and Research Thiruvananthapuram (IISER TVM), Vithura, Thiruvananthapuram 695551, Kerala, India; 5 Department of Information Technology, College of Computer and Information Sciences, Princess Nourah bint Abdulrahman University, P.O. Box 84428, Riyadh 11671, Saudi Arabia; hfmostafa@pnu.edu.sa; 6 University Centre for Research and Development, Chandigarh University, Gharuan, Mohali 140413, Punjab, India; bhawna.e9242@cumail.in

**Keywords:** cervical cell, drift-step rectifier diode, DSRD, reversible electroporation, RE, transmembrane voltage, TMV, viscoelastic

## Abstract

This paper creates an approximate three-dimensional model for normal and cancerous cervical cells using image processing and computer-aided design (CAD) tools. The model is then exposed to low-frequency electric pulses to verify the work with experimental data. The transmembrane potential, pore density, and pore radius evolution are analyzed. This work adds a study of the electrodeformation of cells under an electric field to investigate cytoskeleton integrity. The Maxwell stress tensor is calculated for the dispersive bi-lipid layer plasma membrane. The solid displacement is calculated under electric stress to observe cytoskeleton integrity. After verifying the results with previous experiments, the cells are exposed to a nanosecond pulsed electric field. The nanosecond pulse is applied using a drift-step rectifier diode (DSRD)-based generator circuit. The cells’ transmembrane voltage (TMV), pore density, pore radius evolution, displacement of the membrane under electric stress, and strain energy are calculated. A thermal analysis of the cells under a nanosecond pulse is also carried out to prove that it constitutes a non-thermal process. The results showed differences in normal and cancerous cell responses to electric pulses due to changes in morphology and differences in the cells’ electrical and mechanical properties. This work is a model-driven microdosimetry method that could be used for diagnostic and therapeutic purposes.

## 1. Introduction

Reversible electroporation (RE) occurs when a pulsed electric field creates pores in the plasma membranes of biological cells, which then reseal after some time, without inducing apoptosis or cell death. This method is used to deliver drugs, dyes, and genes into cells. Nanosecond pulsed electric fields have previously been applied to biological cells in reversible electroporation applications [1,2,3]. Many works have used numerical models to understand the electroporation process and the effects of nanosecond pulse strength on biological cells [3,4,5]. These numerical models have considered the biological cells as dispersive models using Debye’s second-order dispersive expression to show the change in dielectric strength with respect to the high-frequency electric pulse [6,7,8,9]. The use of dispersive models has enabled reductions in the strength of the electric field and more accurate predictions of the strength and duration of the electric field required to electroporate cells [10,11,12,13]. In order to make the models more realistic, the two-dimensional morphology of the original biological cells extracted using image processing algorithms has been used to demonstrate the model of electroporation [14,15,16]. These studies supported the reversible electroporation method but advocated using three-dimensional models for more accurate predictions [17,18,19]. The problem of infinite long columns was found to be a limitation when numerically studying all two-dimensional biological cells, whether simple geometries or contours extracted via image processing methods [14]. A realistic three-dimensional model provided more accurate predictions, as demonstrated in [14]. In a previous study, reversible electroporation of a two-dimensional numerical model for all types of cervical cells (superficial, intermediate, parabasal, and basal) was studied [20]. Cervical cells have also been experimentally exposed to electric pulses to achieve electroporation. Cancerous cervical cells have been exposed to a range of pulses with different durations and repetition rates. Electric fields ranging from 0.5 to 5 kV/cm and durations of 25–100 μs rectangular pulses have been used for the electroporation of cervical cells [21]. Another study used Gaussian pulses with a strength of 25 kV/cm and a duration of 200 ns delivered 25 times at 1 Hz repetition rates to achieve irreversible electroporation for cellular apoptosis [22,23,24]. A computational model of the cervical cell, which helps to estimate the strength and duration of the electric field in RE, was not utilized in the above studies.

Cervical cells are squamous epithelial cells. An electric impedance study was conducted on normal and cancerous cervical cells, in which a numerical study was carried out using a cuboid and the dimensions of the cervical cells were accurately measured. The precancerous stages are known as cervical intraepithelial neoplasia (CIN). The three stages of CIN are CIN1, CIN2, and CIN3 [25,26]. In another study, a squamous epithelium cell model was built using a scutoid, prism, and frustum [27,28,29]. Thus, through these studies, it can be stated that cervical cells are flat in nature. The dimensions of cervical cells play an important role in identifying cancerous cervical cells. The dimensional changes in these cells are also an important marker of cancer progression. Another remarkable change observed during cervical cancer stages is the change in the conductivity of the cytoplasm, which becomes more conductive by a factor of 1.8 due to the increased concentration of NaCl [30,31,32]. The cervical cells’ mechanical properties also change as the membrane becomes weak and the elasticity of the cells decreases [33,34,35]. The study and research of electroporation with current electroporators are costly, and the desired high-frequency range electric fields are not available for supra-electroporation [36,37,38]. Nanosecond Gaussian pulses can be used to electroporate cervical cells, as stated in [22,29]. Many pulse generators can be explored and built to deliver these Gaussian pulses. For example, in a previous study, a Marx generator was used to generate Gaussian pulses that were exposed to biological cells [31,33]. A DSRD diode with fast switching abilities has also been used to generate high-voltage and nanosecond Gaussian pulses [32,34,35].

The electrodeformation of cells during electroporation is studied to examine the cytoskeleton integrity of the cells, which is an important condition in RE [37,38,39]. The cells undergo cytolysis due to exposure to high electric pulses. The electrodeformation of biological cells has previously been studied, and multiphysics has been used to inspect cytolysis. Two-dimensional models representing the double-layer membrane as a viscoelastic layer have been studied for CHO cells, yielding accurate results [40]. In previous works, cervical cells underwent deformations during the application of electric fields of low and high frequencies, but a spherical-shaped cell was used in the computational work [41,42,43]. Cervical cells are squamous epithelial cells, and most works have used cuboids and scutoids to demonstrate the cell dimensions. In [36], cuboids were used to represent cells. The authors also studied the dimensions of the normal and cancerous cervical cells. In [41], the authors used a sphere as a 3D model of cervical cells to study electrodeformation. The work carried out in [36] used a frustum and scutoids to represent cell geometry.

Our model uses the original 2D morphology, extrudes the plasma membrane boundaries, and constructs a three-dimensional image. The extrusion depends on the thickness measurement provided by earlier works [25,26]. The nucleus is considered an ellipsoid, and the dimensions are created according to the literature. This model could be used in an initial electroporation study of cervical cells in model-driven microdosimetry. The method involves first extracting the image contour using image processing methods. The images of the normal and cancerous cervical cells are taken from the Herlev database [44]. The cells are then converted into three-dimensional solid images using CAD tools. We first attempt to verify the model with experimental work [21]. A 2.5 kV/cm electric field rectangular pulse of 100 μs is applied to the computational domain, as used in the literature. The cervical cells are then exposed to electric fields with low- and high-frequency single pulses. The TMV, pore density, pore radius evaluation, strain energy, and solid displacement are calculated. The DSRD diode-based electrical circuit is used to generate high-voltage and high-frequency Gaussian pulses. The Pspice tool is then used to simulate the circuit, and the output pulse is then imported into COMSOL 5.5a. The Laplace equation and the Smoluchowiski equation are used to calculate the TMV and pore density. The Debye second-order dispersive equation is used during high-frequency pulses to demonstrate the dispersive nature of cervical cells. The Maxwell stress tensor (MST) is calculated for the cells. The solid displacement is calculated for the viscoelastic plasma membrane. The Young’s modulus of the cancerous cell lowers with cancer progression, thus causing weakening of the plasma membrane and cytoplasm and providing more displacement with applied electric force. The conductivity of cancerous cells is higher, thus causing higher and faster generation of the TMV and pores in the cell. This work, therefore, provides a computational model of normal and cancerous cervical cells and also provides a testing platform for the electroporator before the experimental work.

## 2. Materials and Methods

### 2.1. Three-Dimensional Construction of Normal and Cancerous Cervical Cells

**Figure 1:**
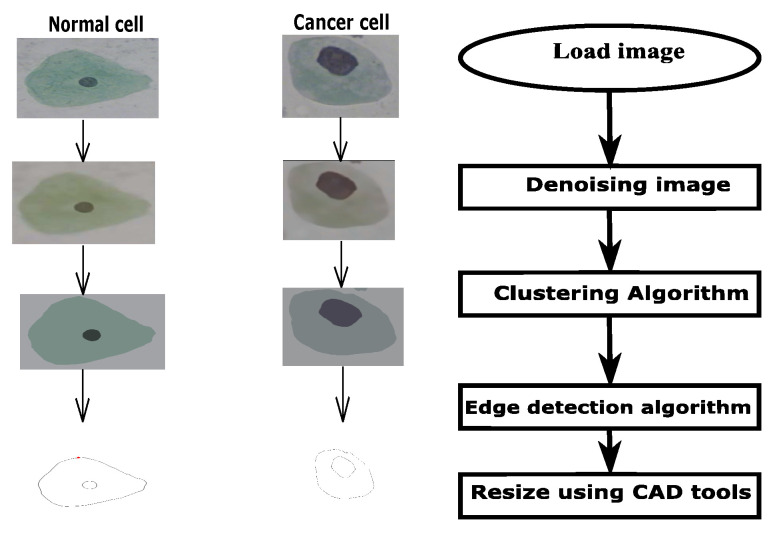
Flowchart for the extraction of contours from the Pap smear images.

**Figure 2:**
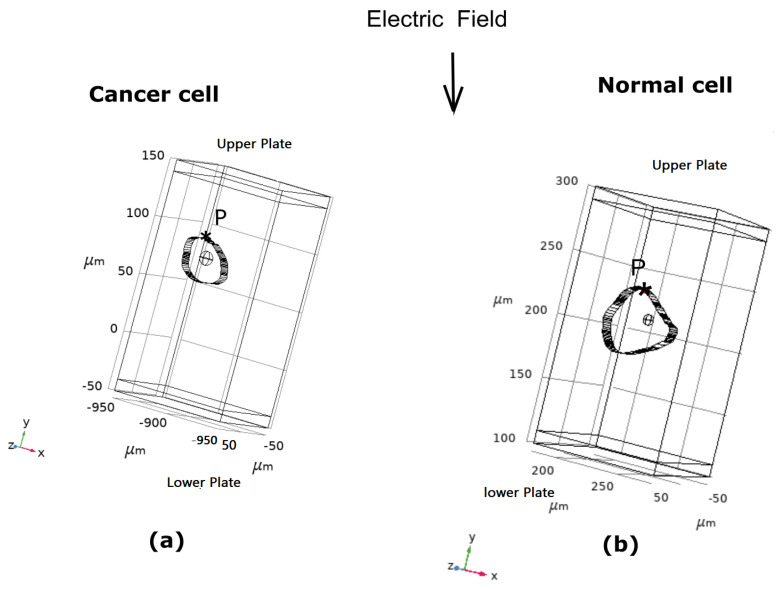
Three-dimensional view of cervical cells in a multiphysics environment, with copper plates on the uppermost plate and the lower plate grounded: (**a**) Containing cancerous cervical cells and (**b**) containing normal cervical cells.

The method described here involves three stages for obtaining the desired results in electroporation. The first stage consists of extracting the contours from the cervical cell images [44]. Figure 1 shows a flowchart of the method used to extract the cell contours and introduce them into COMSOL 5.5. The cervical cells used to demonstrate our work are intermediate (standard cells) and CIN2 (cancer cells). A filter and K-means clustering are used to denoise the images and separate the background from the cytoplasm and nucleus regions. Canny edge detection is used to extract the contours. CAD tools are used to reshape the cell into the size of an intermediate cervical cell. Then, a 3D model is created using extrusion, and an ellipsoid nucleus is placed at the center, using the dimensions presented in the literature [25,26]. Table 1 shows the dimensions of normal (intermediate) cervical cells and the dimensions of CIN1, CIN2, and CIN3 intermediate cells. The image of the CIN2-stage intermediate cells was obtained from the Herlev database, which contains models of cancer cells, and an intermediate normal cell was chosen for our work. Figure 2 shows the orientation of the applied electric field, along with the cancer cell model (Figure 2a) and the normal cell (Figure 2b). The computational domain has dimensions of 200 μm × 100 μm × 100 μm. Here, P indicates the point where the analysis of electroporation was performed.

### 2.2. Electroporation Model of Cervical Cells

#### 2.2.1. Modeling of Cervical Cell Permittivity

The dielectric properties of cervical cells are modeled using a multi-relaxation Debye-based relationship. The dielectric properties of the cervical cells use a dispersive medium, which is modeled using a second-order equation [8].

(1) $$\epsilon \left(\omega \right)-\epsilon_{\infty }=\frac{\Delta \cdot \epsilon_{s}+j\omega \left(\tau_{1}\Delta \cdot \epsilon_{2}+\tau_{2}\Delta \cdot \epsilon_{1}\right)}{1-\omega^{2}\tau_{p}+j\omega \tau_{s}},$$

(2) $$\Delta \epsilon_{s}=\Delta \cdot \epsilon_{1}+\Delta \cdot \epsilon_{2},\tau_{p}=\tau_{1}\tau_{2},\tau_{s}=\tau_{1}+\tau_{2},$$

where $\epsilon_{\infty }$ denotes high-frequency permittivity, $\tau_{1}$ and $\tau_{2}$ are the relaxation times, and $\Delta \epsilon_{1}$ and $\Delta \epsilon_{2}$ are the relaxation amplitudes. The polarization *P* is expressed as a time-varying electric field and is given by:

(3) $$\tau_{p}\frac{d^{2}P}{dt^{2}}+\tau_{s}\frac{dP}{dt}+P=k_{2}\frac{d^{2}E}{dt^{2}}+k_{1}\frac{dE}{dt}+k_{0}E,$$

$$\begin{array}{c} k_{0}=\Delta \cdot \epsilon_{s}+\epsilon_{\infty }-\epsilon_{0},k_{1}=\tau_{1}\left(k_{0}-\Delta \cdot \epsilon_{1}\right)+\tau_{2}\left(k_{0}-\Delta \cdot \epsilon_{2}\right),k_{2}=\tau_{p}\left(k_{0}-\Delta \cdot \epsilon_{2}\right) \end{array}$$

#### 2.2.2. Cervical Cell Pore Formation during Electroporation

Pores with a radius of $r_{p}$ = 0.8 nm are established across the bi-lipid membrane layer due to the nanosecond pulsed electric field. The creation of pores is modeled using an asymptotic Smoluchowski equation:

(4) $$\frac{\partial N}{\partial t}=\alpha e^{{\left(\frac{tmv}{V_{ep}}\right)}^{2})}\left[1-\frac{N}{N_{0}}e^{\left(-q{\left(\frac{tmv}{V_{ep}}\right)}^{2}\right)}\right],$$

where *N* is the pore density on the plasma membrane, $V_{ep}$ is the characteristic voltage of electroporation, α and *q* are the electroporation parameters, and $N_{0}$ is the initial pore density. The transmembrane potential increases as the average conductivity of the cell membrane increases with time, which is updated in the Smoluchowski equation at every step. It leads to an increase in the number of pores. The average conductivity at different pore formation regions is given by

(5) $$\sigma \left(x,y,t\right)-\sigma_{0}=\pi \sigma_{p}r_{p}N\left(x,y,t\right)K_{c},$$

where $K_{c}$ is a constant and can be calculated using Equation (6).

(6) $$K_{c}=\frac{e_{m}^{\upsilon }-1}{\frac{w_{0}.e^{w_{0}}-\eta .\upsilon_{m}}{w_{0}-\eta .\upsilon_{m}}-\frac{w_{0}.e^{w_{0}}+\eta .\upsilon_{m}}{w_{0}+\eta .\upsilon_{m}}},$$

where $w_{0}$ is the pore energy barrier, η is the pore relative entrance length, and $\upsilon_{m}=\frac{q_{e}}{KT}.v_{m}$ is the compensated transmembrane voltage.

#### 2.2.3. Electromagnetic Modeling of Cervical Cells

The electric potential is determined by:

(7) $$\nabla \cdot \nabla \left(\sigma +\epsilon_{0}.\frac{\partial \phi }{\partial t}\right)-\frac{\partial \nabla \cdot P}{\partial t}=0,$$

(8) E=−∇·ϕ,

The transmembrane voltage (TMV) is determined using a special boundary condition, and the cell membrane bi-lipid layer is replaced with a distributed impedance layer, which reduces computational time and space.

(9) $$TMV=\phi_{outer}\left(x,y,t\right)-\phi_{inner}\left(x,y,t\right),$$

(10) $$n.J=\frac{\sigma_{m}}{d_{m}}.\left(V-V_{ref}\right)+\frac{\epsilon_{m}}{d_{m}}.\left(\frac{\partial V}{\partial t}-\frac{\partial V_{ref}}{\partial t}\right),$$

where $V_{ref}$ is the exterior surface potential, and *V* is the interior surface potential of the cell boundary. $d_{m}$, $\sigma_{m}$, and $\epsilon_{m}$ are the thickness, conductivity, and permittivity of the cervical cell membrane. The transmembrane potential is updated at each time step. Smoluchowski’s equation is solved using the weak form of the PDE, and the polarization vector equation is solved using the coefficient form of the PDE interface. At each time step, *P* and $\sigma_{m}$ are updated. The 3D model is solved in an iterative manner. The number of generated finite elements is fewer in number. The development of the pore radius with respect to the time is given by Equations (11) and (12).

(11) $$\frac{dr_{j}}{dt}=\frac{D}{k_{b}T}\left(\frac{V_{m}^{2}F_{max}}{1+r_{h}/\left(r+r_{t}\right)}+4\beta {\left(\frac{r*}{r}\right)}^{4}\frac{1}{r}-2\pi \gamma +2\pi N\delta_{eff}\right),$$

where $V_{m}$ is the generated transmembrane potential. The number of hydrophilic pores developed (*r* > r*) corresponds to *j* = 1, 2, 3, …, $k_{p}$, where $k_{b}$ represents the Boltzmann constant. $\delta_{eff}$ is calculated as:

(12) $$\delta_{eff}=2\delta^{\prime }-\frac{\left(2\delta^{\prime }-\delta_{0}\right)}{{\left(1-\frac{A_{p}}{A}\right)}^{2}},$$

where *A* is the area of the plasma membrane, and the total perforation area is given by $A_{p}$, which is calculated as [7]:

$$\begin{array}{c} A_{p}={∯}_{S}N\left(t\right)\pi r_{j}^{2}dS, \end{array}$$

where *S* is the plasma membrane surface.

#### 2.2.4. Electrodeformation Model of Cervical Cells

The electroporation electrical force is determined using the Maxwell stress tensor (MST):

(13) $$\frac{MST}{\epsilon (t)}=\left(E_{i}.E_{j}-\frac{\delta_{ij}E^{2}}{2}\right),$$

where *E* is the electric field in the *x*-, *y*-, and *z*-directions, ϵ(t) is the dielectric permittivity, and $\delta_{ij}$ is the Knoreckner delta. We have assumed the generalized Maxwell model as the viscoelastic model. The electrical stress is coupled with the mechanical stress on the cervical cell. The structural model membrane displacement *u* is calculated. The membrane is modeled as a viscoelastic model and the remainder of the cell as an elastic model. The strain energy is calculated by first solving

(14) $$\rho \frac{d^{2}u}{dt^{2}}=\delta \times S,$$

where *S* denotes the stress. After obtaining *S*, the strain energy of the deformable cell membrane is calculated as

(15) $$E_{mem}=\int \int \int S\times \gamma d\Omega /2,$$

where *d*Ω is the volume of the element and γ represents the strain.

#### 2.2.5. Temperature Development during RE

The changes in the electroporation temperature are measured using the Pennes bioheat equation [45,46,47], defined as:

(16) $$\nabla \cdot \left(k\nabla T\right)+{\sigma |\nabla \psi |}^{2}+q^{\prime \prime \prime }-W_{b}c_{b}T=\rho c_{p}\frac{\partial T}{\partial t},$$

where *T* is the temperature (310.15 K); $W_{b}c_{b}T$ and $q^{\prime \prime \prime }$ are the heat generated in the bloodstream and metabolism, respectively; $c_{p}$ is the heat capacity; *k* is the thermal conductivity; ψ is the potential developed; and σ is the conductivity. This equation is used to observe whether the developed thermal stresses exceed the safety threshold. Table 2 contains the parameter values used in the computational study.

#### 2.2.6. High-Frequency Electric Field Using DSRD Generator Design

**Figure 3:**
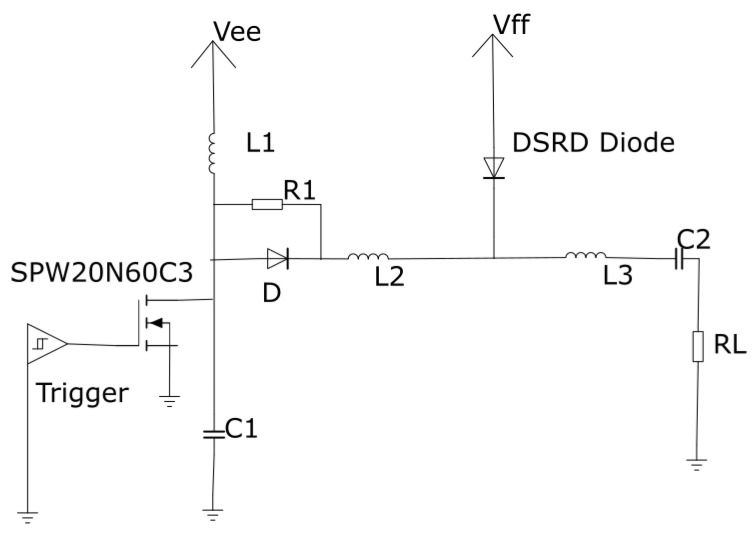
Pulse generator circuit diagram using DSRD diode [34].

The electric field is applied to the copper plates on the top, and the lower part is grounded. The electric field is generated using the simulation of the pulse generator using a drift-step rectifier diode (DSRD), as shown in the circuit diagram in Figure 3. Drift-step rectifier diodes are fast-switching high-voltage devices. In the initial stage, the MOSFET is turned on, allowing a current to flow through coil $L_{1}$. The duration of the current flow is represented by Δt, and its magnitude is determined by the input voltage $V_{ee}$. The charging current passing through coil $L_{1}$ depends on both Δt and $V_{ee}$. Additionally, in the forward direction, the charge passing through the DSRD is influenced by Δt, a voltage $V_{ff}$, and a resistor $R_{1}$. The MOSFET is subsequently switched off, causing the current to reverse its direction through coil $L_{1}$ and flow through the DSRD. The DSRD stays in a reverse conductive mode, allowing the current to flow in the reverse direction. Once the charge from the DSRD is depleted, it practically switches off, allowing the current to flow to the load ($R_{L}$). The load resistance $R_{L}$ determines the magnitude of the current flowing through it. Here, $V_{ee}$ = 18 V and $V_{ff}$ = 11 V in the circuit.

**Figure 4:**
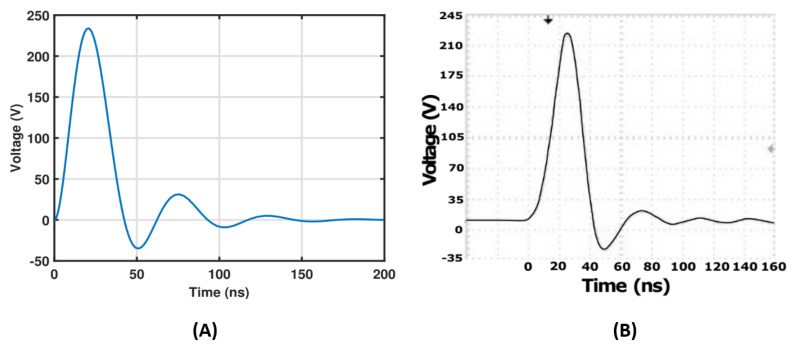
(**A**) Simulation output plot of the DSRD pulse generator. (**B**) Pulse generator output pulse using DSRD diode.

The MOSFET (SPW20N60C3) is activated by the trigger pulse, remaining in an on state through the application of the pulse for a duration of Δ*t* = 50 ns. $V_{ee}$ is applied to determine the charging current passing through $L_{1}$ = 75 nH. The amount of charge passing through the DSRD is determined by Δ*t*, $V_{ff}$, and $R_{1}$ = 20 Ω. When the MOSFET switches off, the resulting current flows in the reverse direction of the DSRD diode. The current flows until the DSRD is in reverse conducting mode. The removal of the charge causes the DSRD to turn off, allowing the current to flow through $R_{L}$ = 50 Ω. $R_{1}$ = 20 Ω plays an important role in balancing the charging of the DSRD in the forward direction. Diode *D* provides the fastest path to discharge $C_{1}$ = 100 pF after the MOSFET switches off. $C_{2}$ = 1 μF and $L_{3}$ = 45 nH are used for impedance matching to the load [34]. The circuit has been simulated in the Pspice environment to obtain the resulting output, as shown in Figure 4A. Figure 4B shows the output response from the pulse generator captured in the digital oscilloscope. The second step is to take this output to the COMSOL 5.5 multiphysics.

## 3. Results

**Figure 5:**
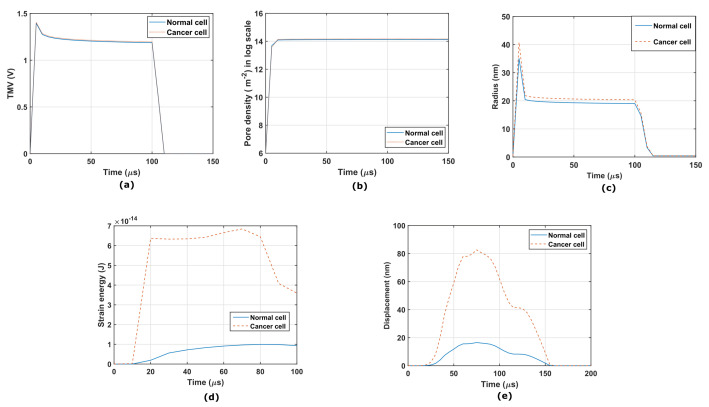
(**a**) Temporal plot of the transmembrane potential of cervical cells at point *P*. (**b**) Temporal pore density (m${}^{-2}$) in log scale at point *P* in cervical cells. (**c**) Temporal plot of the evolution of the pore radius (nm) of cervical cells during RE. (**d**) Temporal plot of the strain energy of cervical cells during RE. (**e**) Solid displacement of cervical cells during electroporation.

The cervical cells are imported into the multiphysics domain with dimensions of 210 μm × 100 μm × 100 μm. The cells are first exposed to a 2.5 kV/cm electric field for a duration of 100 μs. A 1 μs rise and fall time is seen in the rectangular pulse. The copper electrodes are attached to the upper and lower cuboid volumes, as seen in Figure 2a,b. The electric field is applied from the top to the bottom plate, and the lower electrode is grounded. The temporal evolution of the TMV and pore density agrees with our computational work. The temporal pore radius evolution and strain energy density are obtained for a low-frequency pulse. The results agree with the experimental evaluation by reaching the threshold of electroporation. The required TMV (above 1 V) and pore density in log scale (above 14) are generated in normal and cancerous cervical cells, as shown in Figure 5a,b. The pore radius regains its original state after removing the rectangular pulse of 0.8 nm, as shown in Figure 5c. The strain energy ($E_{mem}$) that developed in the cell membrane due to the induced pulses is plotted in Figure 5d. The displacement of the cells is compared to the experimental study, and the deformation can be seen in Figure 5e.

**Figure 6:**
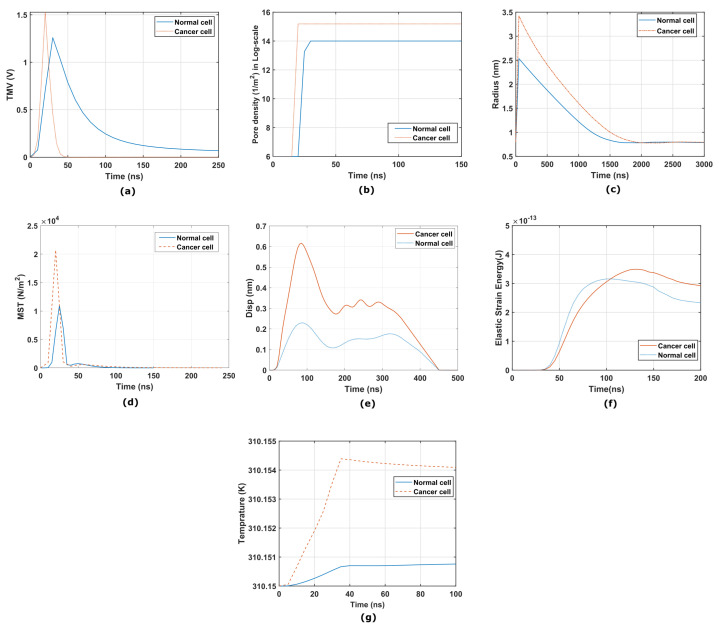
(**a**) Temporal plot of the transmembrane potential of cervical cells at point *P*. (**b**) Temporal pore density (m${}^{-2}$) in log-scale at point *P* in cervical cells. (**c**) Temporal plot of the evolution of the pore radius (nm) of cervical cells during RE. (**d**) Temporal plot of the Maxwell stress tensor (N/m${}^{2}$) of cervical cells at point *P* during RE. (**e**) Solid displacement of the cervical cells during electroporation. (**f**) Temporal plot of the strain energy of cervical cells during RE. (**g**) Temporal plot of the temperature increase during electroporation.

**Figure 7:**
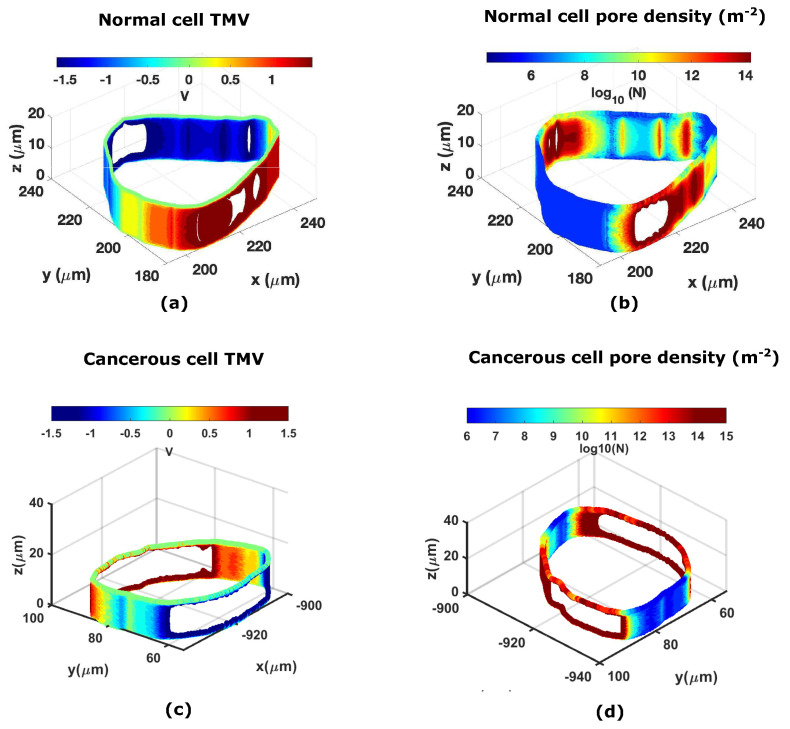
(**a**) Plot of the TMV (V) at 20 ns for normal cervical cells. (**b**) Plot of the pore density in log scale at 20 ns for normal cervical cells. (**c**) Plot of the TMV (V) at 20 ns for cancerous cervical cells. (**d**) Plot of the pore density in log scale at 20 ns for cancerous cervical cells.

The cervical cells are retested under a high-frequency electric field. A Gaussian pulse with a magnitude of 11.5 kV/cm for a duration of 40 ns generated using the above-mentioned method is transferred to COMSOL 5.5a. The temporal evolution of the TMV and pore density is calculated and plotted in Figure 6a,b. The TMV calculation demonstrates that a higher potential developed in cancerous cells compared to normal cells. Similar results can be seen for the temporal development of the pore density and the evolution of the pore radius in cancerous cells (Figure 6c). The development of the Maxwell stress tensor is greater in magnitude and faster in a cancerous cervical cell compared to a normal cervical cell (Figure 6d. Another important observation is that the cervical cells return to their normal state after removing electrical stress, confirming that the membrane integrity is intact. The solid displacement demonstrates this in the cervical cells during electroporation, as shown in Figure 6e. The strain energy ($E_{mem}$) is also calculated for the cervical cells, with cancerous cells developing a greater strain energy. Figure 6f shows the temporal development of the strain energy in the cervical cells. We also checked the increase in the model temperature to prove that RE is a non-thermal process. In Figure 6g, a negligible increase in the temperature can be seen as the initial temperature was maintained at 310 K. Figure 7a depicts a three-dimensional plot of the TMV generated on the surface of a normal cell at 20 ns by the Gaussian pulse when it reaches its peak value. Figure 7b shows the pore density developed at 20 ns. The pore density and TMV reach their maximum values at this time. Similarly, Figure 7b,d depict surface plots of the TMV and pore density generated in the cancerous cells at 20 ns.

## 4. Discussion

The TMV and pore density necessary for RE are generated. The pore radius returns to 0.8 nm, which proves that the model agrees with the results of previous works. Our study adds three-dimensional models of normal and cancerous cervical cells, and the conductivity changes are shown for all the cells. The cancerous cells show a faster generation of the TMV and pore density, as shown in Figure 6a,b, due to the decrease in their sizes, causing more electric fields to develop. This study uses the Pennes bioheat equation to prove that the process is non-thermal. Hence, factors such as the TMV (>1 V), pore density in log scale (>14), and pore radius return to their original size of 0.8 nm, and the non-thermal process is satisfied. Earlier RE numerical models studied the transmembrane potential, pore density, and evolution of the pore radius, which were calculated to explain the physics behind RE. Our work adds the checking of the cytoskeleton integrity of the cervical cells to the study of RE. The application of electric pulses caused a change in the dimensions of the cervical cells. Figure 6e also shows the cervical cells returning to their initial positions after nanosecond electric pulses, from which we observed that no cytolysis was found in the normal or cancerous cervical cells. This study also revealed that, due to differences in the size of the cells, the electrical stress on the cancerous cells was higher compared to the normal cells. Hence, cell dimensions play an important role in electroporation studies.

**Figure 8:**
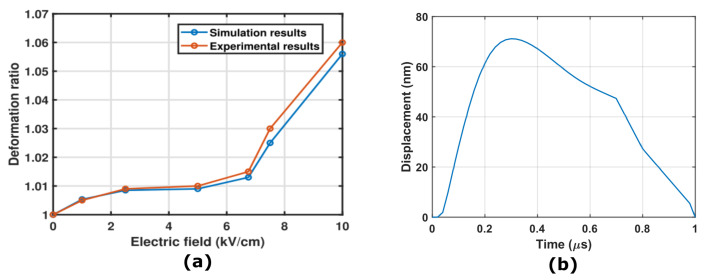
(**a**) Comparison between experimental [41] and simulation results of cervical cells under different electric fields applied for a duration of 1 μs. (**b**) Maximum displacement for the plasma membrane under a 1 kV/cm electric field applied for a duration of 1 μs.

Our simulation revealed a maximum displacement of 72 nm in normal cervical cells, accompanied by an aspect ratio or deformation ratio change (displacement along the *y*-axis/displacement along the *x*-axis) of 1.0053. Figure 8a illustrates a comparison between our model and experimental findings, with a graph relating the applied electric field to the electrodeformation in cervical cells. Notably, our model closely aligns with the experimental results. Figure 8b represents the maximum displacement for the plasma membrane under a 1 kV/cm electric field applied for a duration of 1 μs.

This work can be used for indicative purposes to determine the required strength and duration of electric fields that can be applied to cells. The results could be used in the future to determine whether it is possible to transfer drugs, dyes, and genes into cervical cells before resorting to trial-and-error methods and wasting cell samples.

## 5. Conclusions

This work extracts a two-dimensional image of a cervical cell using simple image processing methods and constructs a three-dimensional geometry of normal and cancerous cervical cells using CAD tools. When exposed to an electric pulse, the three-dimensional cervical cells generated different transmembrane potentials and pore densities due to changes in the dimensions and conductivities of normal and cancerous cells. The cancerous cells showed faster and greater development of the TMV and pore density. This study also examined the viscoelastic movement of the membranes of cervical cells. The elastic properties changed with cancer progression, causing greater solid displacement in cancerous cervical cells. This study provides more insight into cytoskeleton integrity. Thus, in the future, our results could be used to carry out RE experimental studies on cancerous cervical cells at high frequencies. This study could be helpful for diagnostic purposes by detecting cell morphological changes due to cancer progression at an early stage through the influx of dyes into cervical cells. The proposed method could also be used to test drugs and deliver genes into cells. The DSRD generator can potentially be used in the future to deliver dyes into cervical cells to observe morphological changes in the cells due to cancer progression.

## Figures and Tables

**Table 1:** Dimensions of cervical cells.

Cell Type	Cell			Nucleus		
	**X**	**Y**	**Z**	**X**	**Y**	**Z**
Intermediate	52 ± 10 μm	52 ± 14 μm	15 ± 1.1 μm	8.4 ± 1.1 μm	8.4 ± 1.1 μm	5.8 ± 1.4 μm
CIN1	41 ± 10 μm	41 ± 10 μm	14 ± 1.2 μm	7.3 ± 2.2 μm	7.3 ± 2.1 μm	5.4 ± 3.3 μm
CIN2	25 ± 6 μm	25 ± 6 μm	17 ± 1.0 μm	8.1 ± 1.7 μm	8.1 ± 1.7μm	6.9 ± 1.7 μm
CIN3	18 ± 3.2 μm	18 ± 3.2 μm	16 ± 1.8 μm	9.3 ± 1.7 μm	8.6 ± 1.7 μm	8.6 ± 1.7 μm

**Table 2:** Supplementary data.

$\epsilon_{\infty }$	13.9 pF m${}^{-1}$	High-frequency permittivity [11]
$\epsilon_{0}$	8.85 pF m${}^{-1}$	Dielectric permittivity of vacuum [11]
$\epsilon_{r}^{ex}$	72	Relative permittivity of $E_{x}$ medium [25,26]
$\epsilon_{r}^{Cp}$	86	Relative permittivity of $C_{p}$ medium [25,26]
$\epsilon_{r}^{Np}$	145	Relative permittivity of $N_{p}$ medium [25,26]
$\sigma_{Ex}$	1.2 S m${}^{-1}$	Conductivity of $E_{x}$ medium [25,26]
$\sigma_{0}$	9.5 nS m${}^{-1}$	Passive conductivity of $P_{m}$ [11]
$\sigma_{Cp}$	0.6 S m${}^{-1}$	Conductivity of $C_{p}$ [25,26]
$\sigma_{Np}$	0.8 S m${}^{-1}$	Conductivity of $N_{p}$ [25,26]
*q*	2.46	Electroporation constant [11]
$V_{eq}$	224 mV	Characteristic voltage of electroporation [11]
α	$10^{9}$ $m^{-2}s^{-1}$	Pore-creation density [11]
$N_{eq}$	$3.3\times 10^{6}$ m${}^{-2}$	Equilibrium pore density [11]
*h*	5 nm	Plasma membrane thickness [40]
$w_{0}$	3.2	Energy barrier inside the pore [40]
*T*	295 K	Temperature
η	0.15	Relative length of pore entrance area [11]
ρ	1050	Uniform density for all regions [40]
$Y_{ex}$	1000 Pa	Young’s modulus for extracellular medium [40]
$Y_{c}$	500 Pa	Young’s modulus for normal cell cytoplasm [36]
$Y_{mem}$	500 Pa	Young’s modulus for normal cell plasma membrane [36]
$Y_{c}$	300 Pa	Young’s modulus for cancer cell cytoplasm [36]
$Y_{mem}$	300 Pa	Young’s modulus for cancer cell plasma membrane [36]
$v_{ex}$	0.4	Poisson ratio for extracellular medium [40]
$v_{c}$	0.4	Poisson ratio for cytoplasm [40]
$v_{mem}$	0.4	Poisson ratio for plasma membrane [40]
$S_{mem}$	1500 Pa	Branch shear modulus of membrane [40]
*t*	0.1 s	Branch viscous relaxation time of membrane [40]
$K_{E}$	0.41 W/mK	Thermal conductivity of extracellular fluid [45]
$C_{E}$	3780 J/kg-K	Heat capacity at a constant pressure of the extracellular fluid [45]
$K_{C}$	0.6 W/mK	Thermal conductivity of the cytoplasm [45]
$C_{C}$	4718 J/kg-K	Heat capacity at a constant pressure of the cytoplasm [45]
$K_{N}$	0.3 W/mK	Thermal conductivity of the nucleus [45]
$C_{N}$	3000 J/kg-K	Heat capacity at a constant pressure of the nucleus [45]

## Data Availability

Data are contained within the article.

## Abbreviations

The following abbreviations are used in this manuscript:

TMV	Transmembrane voltage
MST	Maxwell stress tensor
RE	Reversible electroporation
DSRD	Drift-step rectifier diode
